# Diversifying the health workforce in Israel and the United States: a comparison

**DOI:** 10.1186/s13584-025-00694-4

**Published:** 2025-06-02

**Authors:** Edward Salsberg

**Affiliations:** The Fitzhugh Mullan Institute for Health Workforce Equity, George Washington University, Washington, DC USA

**Keywords:** Health Workforce, Diversity, Underrepresented Minorities, Arab Israelis, Health Professions Education

## Abstract

Minority populations in many countries, including Israel and the US, experience significant health disparities compared to the majority population. A health workforce that reflects the characteristics of the population including race/ethnicity, language and socioeconomic backgrounds, can help address these disparities. Over the years, Isreal and the US have implemented a variety of programs and policies to promote greater representation of minority populations in medicine and other health professions. This paper compares some of the efforts and outcomes in the 2 countries to support a more diverse health workforce. While progress has been made in both countries, they now face significant challenges which are likely to put recent progress at risk.

## Health workforce diversity in Israel and the US

As a senior health services researcher focused on issues related to underrepresentation of racial and ethnic minorities in the US health professions, I read the paper by Bruce Rosen and Sami Miaari “*Arab Representation in Israeli Healthcare Professions: Achievements*,* Challenges and Opportunities*” [1] with great interest. The paper documents the representation of Arab Israelis in four health professions: medicine, dentistry, pharmacy and nursing, both in the Israeli workforce and in the educational pipeline in Israel. Despite major differences in health care and educational policies and systems, I was struck by both similarities and the differences between the US and Israel in their efforts and challenges to build a diverse workforce reflective of the diversity of their populations.

In both countries, minority populations have historically been underrepresented in the health professions that require advanced education. This reflects numerous factors, including inferior primary and secondary education in minority and lower income communities, the high cost of higher education, limited role models in lower-income communities and racial discrimination, just to name a few.

In the US the lack of a diverse workforce has been documented to contribute to the poorer health status of minority populations. Studies have shown that a workforce concordant with the language, culture and background of the population being served can make a difference in access, quality of care and health outcomes [2-6]. Moreover, a more diverse student body can increase the cultural competence of all medical school graduates [7, 8].

As noted by Rosen and Miaari, health professions education is also an important pathway to economic advancement by providing access to stable, well-paid careers. This is true in both countries, whereby minority health care workers can improve their socioeconomic status for themselves and their families. Arab Israelis have made significant progress in joining the ranks of health care professions. According to Rosen and Miaari while Arab Israeli’s were 22% of the working age Israeli population in 2023, among the active workforce in 2022-23 they were 25% of the physicians, 27% of the dentists, 27% of the nurses and 49% of the pharmacists [1]. This representation was well above their representation just a decade earlier.

In the case of medicine, the greater representation was due to an increase in Arab Israelis educated outside of Israel. As reported by Rosen and Miarri the percent of students in Israeli medical schools who were Arab among all first-degree students in medicine decreased from 18% in 2012 to 9% in 2022 [1]. Even considering the growth in Israeli medical school slots, this reflects a decrease in the number of Arab Israeli graduates from Israeli medical schools. Rosen and Miarri also report that Arab Israelis represented 46% of all newly licensed physicians in 2022. This reflects the high number of Arab Israelis educated in non-Israeli medical schools.

Unfortunately, this educational pathway for physicians is likely to be severely constricted by the Yatziv reform which sets new standards for non-Israeli medical schools with the impact expected to begin in 2026^9^. The failure to significantly increase educational opportunities for Arab Israelis educated within Israel could lead to long term underrepresentation of Arab Israelis in medicine as Arab Israeli’s were as noted above only 9% of the graduates of Israeli medical schools in 2022.

In the US, graduates of foreign medical schools, known as international medical school graduates (IMGs), have been a major source of new physicians (about 25%) each year for decades. While many IMGs have been persons of color including many physicians from the Indian sub-continent, they are not considered underrepresented minorities in medicine. Few IMGs are Black/African Americans, Hispanic/Latino or American Indian, the main population groups considered underrepresented in medicine in the US.

Despite many efforts by the US medical education community to increase access to medical schools for underrepresented groups, progress has been limited. In recent years, Black Americans were 11.5% of the working age population but only 5.7% of the physician workforce and 6.4% of the medical school graduates [9]. In academic year 2024-25, 8.8% of the new MD school entrants were Black students [10]. Hispanic Americans were 18% of the working age population but only 7.4% of the physician workforce and 8.0% of the medical school graduates [9]. In academic year 2024-25, Hispanic students were 11.2% of all new MD matriculants [10].

As noted above, underrepresentation appears to reflect numerous factors including socio-economic differences and educational inequalities. These disparities also appear to reflect the selection criteria and admission processes used by health professions schools that may unintentionally disadvantage low income and minority applicants. Although efforts to diversify health professions school enrollment significantly increased after the 2020 murder of George Floyd, the US Supreme Court banned the consideration of race in school admissions in 2023. This decision appears to have significantly hampered efforts to diversify the workforce. Data from the American Association of Medical Colleges (AAMC) indicates that starting in academic year 2020-21, the annual percentage of entrants into medical schools (MD) that were underrepresented minorities in the US was 22.0%, 24.2%, 22.7%, 23.0% and 20.2% in 2024-25. The changes in Black and Hispanic enrollment dropped by approximately 11% in 2024 compared to 2023 [10].

## Next steps

Given the challenges that minority populations face across the globe, both public and private entities need to continue to find ways to increase educational and job opportunities for health-related professions, particularly for minority populations. Strategies should reflect a broad portfolio of efforts.

### Expansion of educational capacity

The growing demand for health care professionals and expected shortages in both countries should prompt policy makers to increase educational capacity; this has been happening in both countries in recent years and continued growth is expected. Combining growth with some of the strategies described below can significantly increase minority representation in health care.

In 2004, the AAMC called for a major increase in US medical school capacity. This contributed to a major increase in medical (MD) and osteopathic physician (DO) education capacity. While an increase in medical education slots does not guarantee an increase in underrepresented minorities, it is an opportunity to increase overall supply and ease access to medical education for all applicants. Over the 20-year period from 1997 to 2017, the number of entering US medical (MD) students in medicine increased 54%. Although the percentage of underrepresented students decreased (from 15 to 13%), the actual number of underrepresented entering students increased 30% [11].

### Pathway programs

One effective strategy is the development of pathway programs, that is programs that build linkages from educational or other programs before medical school to medical school., For example, specifically designed high school, college or mentorship programs that prepare potential students in the sciences and expose them to careers in medicine. These programs can be designed to fill in educational gaps.

### Admissions criteria

Changes in admissions criteria for medical school is also an important strategy. In the US, several states limited consideration of race in admissions over the past 30 years as did the US Supreme Court for the whole country in 2023. But schools can consider a wide variety of admissions criteria, such as socio-economic factors and other factors associated with success as a physician, in addition to scores on standardized tests in selecting students. Preliminary evaluation provides some evidence that changes in admissions criteria and pathway programs can be effective strategies to support a diverse health workforce [12].

### Support services for medical students

Finally, medical schools can provide support services for all students but designed to meet the needs of non-traditional students, including minority students, that will help students successfully complete their medical education and will also help attract other students in the future.

### Graduate medical education

Policies related to graduate medical education could assist Israeli Arabs educated outside of Israel to assure they are adequately prepared to become licensed physicians especially if there are significant numbers of foreign trained physicians in Israel who have not been able to become licensed.

### Schools targeting underrepresented populations

Another strategy to help diversify the health workforce includes establishing programs specifically designed to attract and retain underrepresented populations. In the US, Historically Black Colleges and Universities (HBCUs) have educated a significant number of Black/African American physicians. Similarly, many osteopathic medical schools located in rural areas have led to a significant increase in enrollment of students from rural communities. Establishing schools in communities with underrepresented populations and implementing programs at those schools for the underrepresented groups can create a welcoming environment and provide an infrastructure supportive of the population.

### Data to inform policies

It is critical to collect and analyze additional data to better examine and monitor the pathways into health professions education and training into practice. This should include data on where students come from, their background, enrollment processes and where graduates go on to practice by race/ethnicity, school, and the population they serve. Surveys of new graduates and at licensure renewal as well as analysis of claims data can enhance our understanding of the physician workforce.

## Conclusion

As noted above, while 9% of newly graduated physicians from Israeli medical schools in 2022 were and Arab Israelis, 46% of newly licensed physicians were graduates of foreign medical schools. This dependence on foreign education for Arab Israelis is problematic since many do not meet current and forthcoming educational standards for licensure. Israel’s implementation of the Yatziv reform, needed to assure well prepared physicians, is likely to reduce the number of new physicians each year, especially Arab Israeli physicians [13, 14]. This is likely to reverse the progress made in diversifying the Israel physician workforce. A major expansion of Israeli medical education capacity combined with other programs will be needed to maintain representation of Arab Israelis in the medical workforce.

Israel and the US are now facing significant additional challenges in efforts to support initiatives for minority populations. Israel faces the enormous challenge of the war with Hamas and the tensions flowing from the war between Arabs and Jews. In the US, we face major challenges given the US Supreme Court banning affirmative action in educational admissions and the new Trump administration’s aggressive actions against programs designed to address underrepresentation. Nevertheless, given the importance and benefits of a workforce reflective of the population being served, broad-based creative efforts to ensure a diverse health workforce must continue.

## Data Availability

No datasets were generated or analysed during the current study.
